# Prevention of Military Epidemics of Cerebro-Spinal Fever

**Published:** 1919

**Authors:** 


**Prevention of Military Epidemics of Cerebro-Spinal Fever.** By Capt. J. A. Glover, R. A. M. C. *Journal of Hygiene*, October 1918, and *British Medical Journal*, November 9, 1918.

The author draws the following conclusions from carefully conducted observations in dealing with the control of epidemic strains of meningococcus in variously housed detachments of the British Army.

A high meningococcus carrier-rate in a military unit denotes dangerous overcrowding. The distance between beds is of paramount importance. Severe overcrowding, i. e., when beds are less than a foot apart, is usually accompanied by a carrier-rate of at least 20 o/o. This is the danger line indicated in the War Office Memorandum on Cerebro-Spinal Fever, March, 1917.

Carrier-rates of between ten and twenty per cent. are unsatisfactory and imply overcrowding. They must be watched with suspicion, and the mobilization standard must be strictly enforced. A carrier-rate of this height will usually imply that the mobilization standard of forty square feet per man has been infringed, and also that beds are less than a foot apart.

A carrier-rate of twenty per cent. should be a signal for prompt action to abolish overcrowding, to improve ventilation, and to increase distance between beds to at least two and one-half feet. The "peace" standard of three feet between beds, and sixty square feet of floor space with six hundred cubic feet of air, would, of course, be more effective.

But the mobilization standard, introduced for a grave emergency, is the lowest possible concession to military necessity which can be allowed with safety.

